# Fluid–Structure Interaction Analysis of Trapezoidal and Arc‐Shaped Membranes Mimicking the Organ of Corti

**DOI:** 10.1002/cnm.3896

**Published:** 2024-12-25

**Authors:** Kentaro Doi, Sho Takeuchi, Hiroki Yamazaki, Tetsuro Tsuji, Satoyuki Kawano

**Affiliations:** ^1^ Department of Mechanical Science and Bioengineering, Graduate School of Engineering Science Osaka University Osaka Japan

**Keywords:** animal test, artificial basilar membrane, fluid–structure interaction, PVDF, resonant frequency, Ringer's solution

## Abstract

In a previous study [H. Shintaku et al., *Sensors and Actuators A: Physical* 158 (2010): 183–192], an artificially developed auditory sensor device showed a frequency selectivity in the range from 6.6 to 19.8 kHz in air and from 1.4 to 4.9 kHz in liquid. Furthermore, the sensor succeeded in obtaining auditory brain‐stem responses in deafened guinea pigs [T. Inaoka et al., *Proceedings of the National Academy of Sciences of the United States of America* 108 (2011): 18390–18395]. Since then, several research groups have developed piezoelectric auditory devices that have the capability of acoustic/electric conversion. However, the piezoelectric devices are required to be optimally designed with respect to the frequency range in liquids. In the present study, focusing on the trapezoidal shape of the piezoelectric membrane, the vibration characteristics are numerically and experimentally investigated. In the numerical analysis, solving a three‐dimensional fluid‐structure interaction problem, resonant frequencies of the trapezoidal membrane are evaluated. Herein, Young's modulus of the membrane, which is made of polyvinylidene difluoride and is different from that of bulk, is properly determined to reproduce the experimental results measured in air. Using the modified elastic modulus for the membrane, the vibration modes and resonant frequencies in liquid are in good agreement with experimental results. It is also found that the resonant characteristics of the artificial basilar membrane for guinea pigs are quantitatively reproduced, considering the fluid–structure interaction. The present numerical method predicts experimental results and is available to improve the frequency selectivity of the piezoelectric membranes for artificial cochlear devices.

## Graphical abstract

In the numerical analysis, solving a three‐dimensional fluid–structure interaction problem, resonant frequencies of the trapezoidal membrane are evaluated. Using the modified elastic modulus for the membrane, the vibration modes and resonant frequencies in liquids are in good agreement with experimental results. It is also found that the resonant characteristics of the artificial basilar membrane for guinea pigs are quantitatively reproduced, considering the fluid–structure interaction.

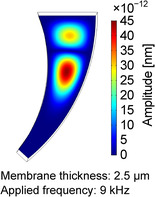

## 1 Introduction

Hearing is one of the most important senses for good communication in human society. Therefore, congenital or aging deafness will cause a serious decline in the quality of life. Sensorineural hearing loss mainly caused by cochlear disease is the most serious, although there may be various reasons for hearing loss [1, 2, 3]. The acoustic/electric conversion mechanism of mammalian ears has been mostly clarified [4]. The ear mainly consists of three parts, such as the external, middle, and inner ears. A sound that propagates through the air is first transmitted to a compressive wave on the eardrum by the external ear and the ear canal. The middle ear, which consists of three small bones, the malleus, incus, and stapes, transmits the eardrum vibration to the oval window of the cochlea. Herein, we especially focus on the function of the cochlea, which consists of three layers filled with lymphatic liquids: the scala vestibuli, scala media, and scala tympani. A vibration of the oval window induces perilymph liquid motion in the scala vestibuli and is compensated by the round window at the end of the scala tympani that is connected to the former at the apex of the cochlea. The scala media, which is filled with the endolymph liquid, is separated from the scala vestibuli by Reissner's membrane and from the scala tympani by the basilar membrane. The mechanosensitive hair cells are embedded in the organ of Corti on the basilar membrane. This suggests that a pressure difference between the top and bottom surfaces of the basilar membrane induces the vibration and lets the mechanosensitive hair cells stimulate the auditory nerves [2, 5, 6]. A sound frequency ranging from 20 to 20,000 Hz can be identified by the basilar membrane because of the tapered shape, which has 500‐μm apical and 100‐μm basal widths and a 3‐mm length [5, 7]. The outer hair cells amplify the amplitude, and the inner hair cell transduces the mechanical vibration to an electrical signal [4].

To remedy sensorineural deafness, the cochlear function is required to be recovered. Artificially developed cochlear devices have already been commercialized [8, 9, 10]. On the other hand, there are some problems that remain to be improved. Recently, piezoelectric artificial basilar membranes, which spontaneously generate electric voltages to directly stimulate auditory nerves, have been developed for the practical use of fully implantable artificial cochlea. A few research groups experimentally succeeded in obtaining electrically evoked auditory brain‐stem responses in deafened guinea pigs by using the next‐generation artificial cochlear device [1, 3]. Shintaku et al. [11, 12] and Tanujaya et al. [13, 14] applied a polyvinylidene difluoride (PVDF) membrane for the artificial basilar membrane, which was a flexible piezoelectric film that was nontoxic to the human body. The trapezoidal shape of the membrane works as an analog spectrum analyzer that performs the Fourier transform [15], and the electrodes integrated on the membrane can immediately stimulate auditory nerves [11, 16]. Lee et al. [17] fabricated a piezoelectric thin film of Pb[Zr_*x*_Ti_1−*x*_]O_3_ (PZT) and used the film to mimic the cochlear basilar membrane. They also reported that the frequency selectivity could be realized by designing a trapezoidal shape. Jung et al. [18, 19] also demonstrated that a multichannel acoustic membrane achieved frequency selectivity and worked as an acoustic sensor for an artificial cochlear device. Jang et al. [20, 21] and Kim et al. [22] fabricated AlN thin films that had piezoelectric responses to sound pressures and developed a micro‐electro‐mechanical device for the artificial cochlea. Reconstruction of the audio input from the artificial cochlear model was also conducted [23]. Malherbe et al. [24] developed a three‐dimensional implanted cochlear model from standard CT scan data and investigated potential distributions and neural excitation patterns. Successively, a novel procedure to design optimal electrode configurations for cochlear implants was proposed [25]. Furthermore, a three‐dimensional finite element model was applied to simulate the effect of endolymphatic hydrops in the basilar membrane on hearing loss [26]. Saremi and Stenfelt [27] revealed the availability of biophysical models to simulate noise‐induced hair cell pathologies. Recently, we succeeded in developing a prototype device that mimicked the amplification function of outer hair cells [28, 29]. Furthermore, the artificial auditory membrane is required to be sensitive to the human audible frequency range from 20 to 20,000 Hz. To achieve such a requirement, the dimensions and materials of the membrane have to be finely tuned.

In the present study, we develop a computational model to analyze the resonant frequency of trapezoidal PVDF membranes in lymphatic liquids. The resonant frequency of PVDF membranes is numerically evaluated using a three‐dimensional finite element method (FEM). Based on the conventional theoretical models [5, 6, 30, 31, 32], it is confirmed that the amplitude of the vibration presents the maximum peak at the resonant position that shifts to the basal side with increasing frequency. This result agrees with a traveling wave with a resonance proposed by Lighthill [6], where a liquid also has an important role in reducing the high frequency of rigid bodies because of the damping and inertia. In the three‐dimensional model, the physical properties of specific thin films are suitably extrapolated from the bulk values. We also conduct experiments to clarify the resonant frequency and its selectivity of the trapezoidal PVDF membrane in liquid, where the membrane displacement induced by the inverse piezoelectric effect is measured with a laser Doppler vibrometer (LDV). In our previous studies, Shintaku et al. [11] reported the frequency selectivity of trapezoidal PVDF membranes ranging from 6.6 to 19.8 kHz in air and from 1.4 to 4.9 kHz in silicone oil, and Inaoka et al. [1] detected the resonant frequency of artificial basilar membranes made of PVDF trifluoroethylene (PVDF‐TrFE) implanted in a guinea pig as 3 and 9 kHz, as summarized in Table 1. Recently, we also reported the resonant characteristics of the strip array of PVDF membranes fixed across a trapezoidal through hole, which had a frequency selectivity in the range from 8.7 to 21.8 kHz in air and from 2.6 to 6.1 kHz in silicone oil. It was clarified that the detectable frequency window was apparently shifted lower in liquid. On the other hand, the effects of the fluid‐structure interaction on the resonant frequency have remained to be clarified. In this study, the numerical analysis above is found to well reproduce the experimental results. Herein, we focus on the vibrations of elastic bodies because PVDF membranes usually show linear elasticity. Although the nonlinear properties of the basilar membrane are known to be an important issue in the auditory mechanism, the unique functions of inner and outer hair cells are complex challenges in the future. It is expected that the present results shed light on the establishment of a numerical procedure for the optimal design of the piezoelectric auditory membrane that is required to achieve human‐audible‐range resonant frequencies.

**TABLE 1:** Resonant frequencies of piezoelectric membranes developed for the artificial basilar membrane.

Shape	Material	Fluid	Membrane thickness (μm)	Resonant frequency (kHz)	
Trapezoidal [11]	PVDF	Air	40	6.6 (min)	19.8 (Max)
Trapezoidal [11]	PVDF	Silicone oil	40	1.4 (min)	4.9 (max)
Curved [1]	PVDF‐TrFE	Lymphatic liquid	3	3 (min)	9 (2nd)
Trapezoidal beam array [15]	PVDF	Air	28	8.7 (min)	21.8 (max)
Trapezoidal beam array [15]	PVDF	Silicone oil	28	2.6 (min)	6.1 (max)

## 2 Materials and Methods

### 2.1 Vibration Analysis of Basilar Membrane in Liquid

In this section, a mathematical model is developed to analyze the vibration characteristics of a trapezoidal membrane in liquid, which is a magnified model of the human cochlea, the coiled shape of which is stretched to clarify its function as a spectrum analyzer. Membrane vibration modes in the air may be simply evaluated by solving a Helmholtz equation of elastic body. On the other hand, in the cochlea, vibrations of the basilar membrane, which are confined between the scala media and the scala tympani, are induced by pressure fluctuations between both liquid layers. As shown in previous reports [2, 5, 6], it was suggested that the propagation of sound pressure in the perilymph liquid causes a weak pressure difference that triggers a vibration of the membrane. A sound pressure applied on the trapezoidal basilar membrane induces a traveling wave that is localized at a resonant point. Although several methods for the vibration analysis of the peculiar membrane were developed [2, 5, 6], the Wentzel–Kramers–Brillouin (WKB) approximation had a great contribution to deepen the understanding of the frequency selectivity of the basilar membrane [11, 33, 34]. In the present study, furthermore, we propose a computational procedure to quantitatively evaluate the resonant frequency of the trapezoidal PVDF membrane exposed to pressure fluctuations in liquids. In such a condition, vibrations should be formulated based on the interaction between the elastic membrane and perilymph liquid, assuming that the artificial basilar membrane will be placed in the scala tympani filled with the perilymph liquid [1].

#### 2.1.1 Vibration of an Elastic Basilar Membrane

In a three‐dimensional space, the vibration of a basilar membrane is expressed by the constitutive equation of an elastic body, as follows [35]:

(1a) $$\rho_{m}\frac{\partial^{2}\zeta_{x}}{\partial t^{2}}=\frac{\partial \sigma_{x}}{\partial x}+\frac{\partial \tau_{\mathrm{yx}}}{\partial y}+\frac{\partial \tau_{\mathrm{zx}}}{\partial z}$$

(1b) $$\rho_{m}\frac{\partial^{2}\zeta_{y}}{\partial t^{2}}=\frac{\partial \tau_{\mathrm{xy}}}{\partial x}+\frac{\partial \sigma_{y}}{\partial y}+\frac{\partial \tau_{\mathrm{zy}}}{\partial z}$$

(1c) $$\rho_{m}\frac{\partial^{2}\zeta_{z}}{\partial t^{2}}=\frac{\partial \tau_{\mathrm{xz}}}{\partial x}+\frac{\partial \tau_{\mathrm{yz}}}{\partial y}+\frac{\partial \sigma_{z}}{\partial z}$$

where $\rho_{m}$ is the density of the elastic body; $\zeta_{i}\left(x,y,z,t\right)$, (i=x,y,z) is the displacement in the i‐axis, $\sigma_{i}\left(x,y,z,t\right)$ is the tensile stress in each principal axis; and $\tau_{\mathrm{ij}}\left(x,y,z,t\right)$, ($\left\{i,j\right\}=\left\{x,y,z\right\}$) is the shear stress on the ij‐plane. According to the relation between stress and strain for isotropic media, $\sigma_{i}$ and $\tau_{\mathrm{ij}}$ are given by [36]

(2) $$\sigma_{i}=E_{\mathrm{ii}}\varepsilon_{\mathrm{ii}}+\sum_{j\neq i}E_{\mathrm{jj}}\varepsilon_{\mathrm{jj}}$$

(3) $$\tau_{\mathrm{ij}}=E_{\mathrm{ij}}\varepsilon_{\mathrm{ij}}\;\text{for}\;j\neq i$$

(4) $$E_{\mathrm{ii}}=\frac{1-\eta }{\left(1+\eta \right)\left(1-2\eta \right)}E$$

(5) $$E_{\mathrm{jj}}=\frac{\eta }{\left(1+\eta \right)\left(1-2\eta \right)}E\;\text{for}\;j\neq i$$

(6) $$E_{\mathrm{ij}}=\frac{E}{2\left(1+\eta \right)}\;\text{for}\;j\neq i$$

where $\varepsilon_{\mathrm{ij}}$ are the strain tensors, E and η are, respectively, Young's modulus and Poisson's ratio which are unique for isotropic materials.

#### 2.1.2 Sound Pressure Propagation in Liquid

Herein, we assume that liquid flows are confined in a narrow space, like a perilymph liquid in the cochlea, and that the viscous term is more dominant than the inertial term, which is a low‐Reynolds‐number condition. In such a situation, the pressure P, liquid density $\rho_{f}$, and flow velocity u fluctuate near an equilibrium state. The pressure P consists of the fluctuation δp and the background pressure $\bar{p}$ such that $P=\bar{p}+\mathrm{\delta p}$. The density $\rho_{f}$ fluctuates ${\delta \rho }_{f}$ near the bulk density ${\bar{\rho }}_{f}$, such that $\rho_{f}={\bar{\rho }}_{f}+{\delta \rho }_{f}$. The flow velocity $u\left(x,y,z,t\right)$ equilibrated is changed by the external forces. Removing the negligibly small values, the mass conservation law is expressed as follows:

(7) $$\frac{\partial {\delta \rho }_{f}}{\partial t}+{\bar{\rho }}_{f}\nabla \cdot u=0$$

In general, the perturbation ${\delta \rho }_{f}$ is zero or negligible within a short time average, and then Equation (7) satisfies ∇⋅u=0, where the liquid behaves as an incompressible flow. Assuming low‐Reynolds‐number conditions, in which liquids are constrained in irrotational motions, the liquid motion is expressed by linearized Navier–Stokes equations, as follows:

(8) $${\bar{\rho }}_{f}\frac{\partial u}{\partial t}=-\nabla \mathrm{\delta p}+\tilde{V}\mu \Delta u$$

where μ is the viscosity of the liquid, $\tilde{V}$ is a constant expressed with an empirical factor defined as the bulk viscosity coefficient $\mu_{b}$ [35], such that

(9) $$\tilde{V}=\frac{4}{3}+\frac{\mu_{b}}{\mu }$$

According to the conventional theory [6], the adiabatic (isoentropic) condition is maintained in the cochlea, and then the pressure depends only on the density:

(10) $$\mathrm{\delta p}=c_{0}^{2}{\delta \rho }_{f}$$

where $c_{0}$ is the speed of sound in the liquid. This kind of condition was also applied to problems of beam vibrations in viscous liquids [32, 37]. Taking the divergence of Equation (8) and removing u using Equations (7) and (10), we obtain

(11) $$\frac{\partial^{2}\mathrm{\delta p}}{\partial t^{2}}-\nu \tilde{V}\Delta \frac{\partial \mathrm{\delta p}}{\partial t}-c_{0}^{2}\Delta \mathrm{\delta p}=0$$

where $\nu =\mu /{\bar{\rho }}_{f}$ is the kinematic viscosity. Equation (11) expresses pressure wave propagation in liquid.

#### 2.1.3 Boundary Conditions of the Fluid–Structure Interaction

For the vibration mode analysis of a membrane in liquid, we need to set a boundary condition to consider the fluid–structure interaction. When a minute vibration occurs, $\mathrm{\delta p}\left(x,y,z,t\right)$ is simply replaced by $\delta p_{\omega }\left(x,y,z\right)\exp\left(\mathrm{i\omega t}\right)$ with an angular frequency ω. In the computational domain, an artificial basilar membrane contacts a fluid at the top and bottom surfaces, $S_{1}$ and $S_{2}$, respectively, and then both media oscillate, contacting at the interfaces. In such a situation, both boundaries have to satisfy

(12) $$\frac{1}{{\bar{\rho }}_{f}}\left(1+\mathrm{i\omega}\frac{\nu \tilde{V}}{c_{0}^{2}}\right)n\cdot \nabla \mathrm{\delta p}=n\cdot \frac{\partial^{2}\zeta }{\partial t^{2}}\;\mathrm{on}\;S_{1}\;\text{and}\;S_{2}$$

where n is the surface normal vector and ζ is the displacement vector. Here, δp and $\zeta ={\left(\zeta_{x},\;\zeta_{y},\;\zeta_{z}\right)}^{T}$ are treated as complex functions, and, finally, the real parts only have physical meaning. The mathematical model described above is numerically solved using FEM methods implemented in commercial software [38]. Details of the other boundary conditions are discussed in the following sections, focusing on vibrations in air and liquid.

### 2.2 Experimental Methods

**FIGURE 1:**
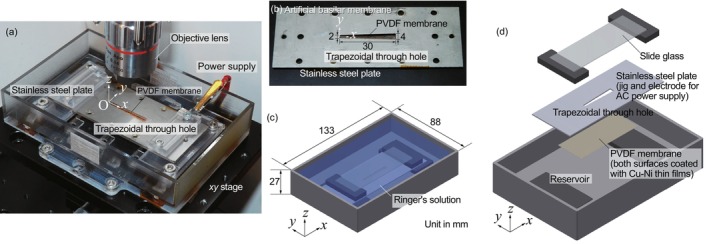
(a) Photograph of the entire view of the experimental device in which a piezoelectric PVDF membrane coated with copper–nickel thin films adheres at the back of a stainless steel plate, which is fixed in a liquid chamber. An AC electric potential is applied to the bottom surface of the piezoelectric membrane that is connected to a lead terminal via an electroconductive adhesive tape. (b) A trapezoidal through hole in a stainless steel plate to maintain a fixed boundary condition of the PVDF membrane, which is the same dimension as the artificial basilar membrane as introduced in a previous study [11]. (c) Schematic view of the experimental apparatus and (d) stacks of each element.

**FIGURE 2:**
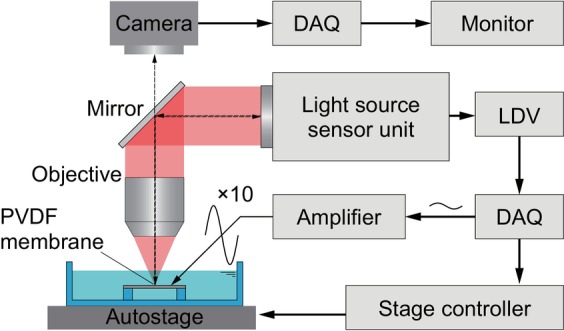
Schematic diagram of the experimental system. An electric potential is 10 times amplified and applied to a PVDF membrane to induce a vibration that is measured by a laser Doppler vibrometer (LDV). The electric power supply, displacement measurement, and motorized stage are controlled by using a data acquisition (DAQ) system.

Herein, we experimentally measure the vibration characteristics of a trapezoidal‐shaped PVDF membrane for the artificial cochlea. Figure 1a shows a stainless steel plate fixed with screws in a liquid chamber, which has a width of 88 mm, a length of 133 mm, and a height of 27 mm, and the position can be adjusted with a motorized xy stage (SGSP26‐150/SGSP20‐35, Sigmakoki Co. Ltd., Tokyo, Japan). As shown in Figure 1b, a trapezoidal through hole is made in the stainless steel plate, which has an upper base of 2 mm, a lower base of 4 mm, and a height of 30 mm in the same dimension as a previous study [11]. This is a magnified straight model of two and a half turns of a basilar membrane in the human cochlea. A rectangular‐shaped PVDF membrane (2‐1003702‐7, TE Connectivity Ltd., Schaffhausen, Switzerland), which has a 25‐mm width, a 60‐mm length, and a 52‐μm thickness, is fixed along the edge of a through hole using double‐side adhesive tape. Both surfaces of the PVDF membrane are coated with copper–nickel alloy thin films for electrodes, and the laminated film is totally 52 μm thick. The dimension and assembly of the experimental device are also schematically shown in Figure 1c,d. An ac electric potential is supplied to a PVDF membrane via an electroconductive adhesive tape touched to a lead terminal, which is connected to a bipolar power amplifier (HSA4014, NF Corp., Yokohama, Japan) to apply an ac electric potential difference of $V_{\text{in}}=V_{0}\sin\left(2\mathrm{\pi ft}\right)$ with an amplitude of $V_{0}$ and a frequency of f, where the other terminal is grounded. The electrically polarized membrane is deformed due to the inverse piezoelectric effect and results in vibrations by the applied ac electric potential. A displacement in the z direction is measured with a laser Doppler vibrometer (LDV) (AT3600, Graphtec Corp., Yokohama, Japan). The focal point of an irradiated laser beam of 633 nm in wavelength is adjusted with the xy stage by monitoring a view camera. The electric power supply and the displacement measurement are controlled using a data acquisition (DAQ) system, which consists of a power supply (NI PXIe‐1082, National Instruments Corp., Austin, USA), a controller (NI PXIe‐8840, National Instruments Corp., Austin, USA), an analog output module (NI PXIe‐6738, National Instruments Corp., Austin, USA), and a sound and vibration module (NI PXIe‐4492, National Instruments Corp., Austin, USA). The entire experimental system is schematically shown in Figure 2. In the present study, an electric potential of 0.2 V peak‐to‐peak is applied by an electric power supply in the DAQ system and is amplified 10 times by the amplifier. The membrane vibration is measured at the center of the minor axis, i.e., y=0mm, along the *x*‐axis. The motorized xy stage shifts the membrane position to the focal point of the laser beam. The sampling frequency and sampling time are set to 10^5^ Hz and 0.1 s, respectively. Measured displacements are immediately translated to the Fourier spectra, and the resonant frequency is analyzed.

## 3 Results and Discussion

First, the present computational methods, mesh size and accuracy, were verified, considering simple problems about a forced oscillation of an elastic beam in a vacuum and that of water in a square duct. Numerical details and results of these problems are presented in Appendix. As a result, the computational errors were less than 1% compared to the theoretical estimations. The mesh size for the FEM analysis was determined to be fine and the numerical results maintained three significant figures. Second, the vibration mode of a trapezoidal PVDF membrane that was placed in a vacuum was analyzed. Based on the validations above, the resonant frequency of the PVDF membrane for the artificial basilar membrane was quantitatively evaluated, considering fluid–structure interactions. The vibration characteristics in the air, silicone oil, and Ringer's solution were compared between the computational and experimental results. Finally, the resonant characteristics of the artificial basilar membrane for guinea pigs [1] were numerically analyzed.

### 3.1 Resonant Frequency Analysis of Trapezoidal PVDF Membranes

**FIGURE 3:**
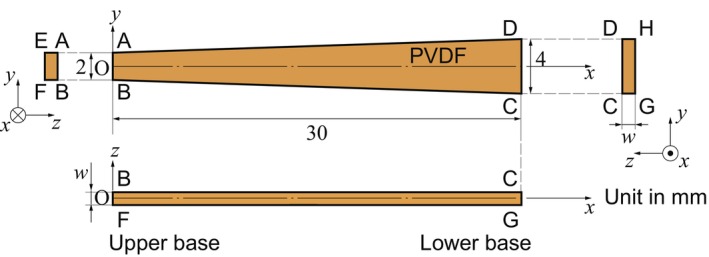
Schematic illustration of a trapezoidal basilar membrane, which has a 2‐mm upper base, a 4‐mm lower base, and a 30‐mm height, fixed at side walls of AEFB, BFCG, CDHD, and DHEA. Uniform stress $\sigma_{z}=p_{0}\sin\left(2\mathrm{\pi ft}\right)$ is applied on the surface of ABCD. In addition, w is set to 52 μm, not to scale.

In a previous study [1, 11], we performed experiments of resonant frequency analysis of trapezoidal PVDF membranes that were fixed on a stainless steel plate. It was suggested that the trapezoidal shape that modeled the basilar membrane in the cochlea was important to geometrically separate the resonant frequencies on a single membrane. Using a trapezoidal PVDF membrane having a thickness of 40 μm, the resonant frequency was varied from 6.6 to 19.8 kHz in air [11]. To construct the theoretical model and numerical methods, herein, we performed FEM analysis of a trapezoidal PVDF membrane. As shown in Figure 3, the resonant frequency of a trapezoidal‐shape elastic body, whose side walls were fixed, was numerically evaluated using the aforementioned procedures. The upper and lower bases of the trapezoid were set to 2 and 4 mm, respectively, and the longitudinal length was 30 mm. The thickness of the membrane was set to w=52μm, which was the membrane used in the experiment of the present study [11]. According to the physical properties of anisotropic PVDF [38, 39, 40, 41], the density and elasticity were respectively set to $\rho_{m}=1.78\times 10^{3}\;\mathrm{kg}\;m^{-3}$ and

(13) $$E=\left(\begin{array}{cccccc} 3.8 & 1.9 & 0.9 & 0 & 0 & 0\\ 1.9 & 3.8 & 0.9 & 0 & 0 & 0\\ 0.9 & 0.9 & 1.2 & 0 & 0 & 0\\ 0 & 0 & 0 & 0.9 & 0 & 0\\ 0 & 0 & 0 & 0 & 0.7 & 0\\ 0 & 0 & 0 & 0 & 0 & 0.9 \end{array}\right)\;\mathrm{GPa}$$

Young's modulus and Poisson's ratio of PVDF were determined as *E* = 2.4 GPa and η=0.35 from previously reported experimental results [39, 40], respectively. The linear relation between the stress and strain was defined as

(14) $$\left(\begin{array}{c} \sigma_{x}\\ \sigma_{y}\\ \sigma_{z}\\ \tau_{\mathrm{yz}}\\ \tau_{\mathrm{zx}}\\ \tau_{\mathrm{xy}} \end{array}\right)=E\left(\begin{array}{c} \varepsilon_{\mathrm{xx}}\\ \varepsilon_{\mathrm{yy}}\\ \varepsilon_{\mathrm{zz}}\\ 2\varepsilon_{\mathrm{yz}}\\ 2\varepsilon_{\mathrm{zx}}\\ 2\varepsilon_{\mathrm{xy}} \end{array}\right)$$

Boundary conditions were

(15) $$\sigma_{z}=p_{0}\sin\left(2\mathrm{\pi ft}\right),\;\mathrm{on}\;\text{ABCD}$$

(16) $$\frac{\partial \zeta_{z}}{\partial z}=0\;\mathrm{on}\;\text{GHEF}$$

(17) $$\zeta_{x}=\zeta_{y}=\zeta_{z}=0\;\mathrm{on}\;\text{AEFB},\;\text{BFGC},\;\text{CGHD},\;\text{and}\;\text{DHEA}$$

A pressure difference of $p_{0}\sin\left(2\mathrm{\pi ft}\right)$ was applied to the top surface (ABCD) relative to the bottom surface (EFGH).

**FIGURE 4:**
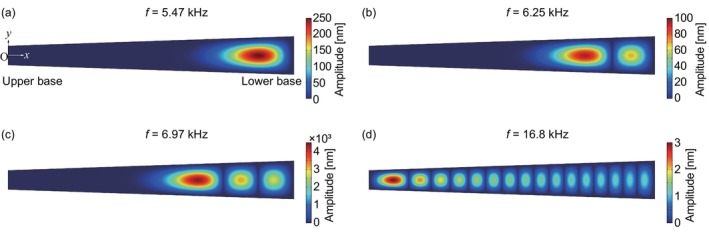
The numerical result of amplitude distributions for some typical eigenfrequencies of the trapezoidal PVDF membrane: (a) 5.47, (b) 6.25, (c) 6.97, and (d) 16.8 kHz.

**FIGURE 5:**
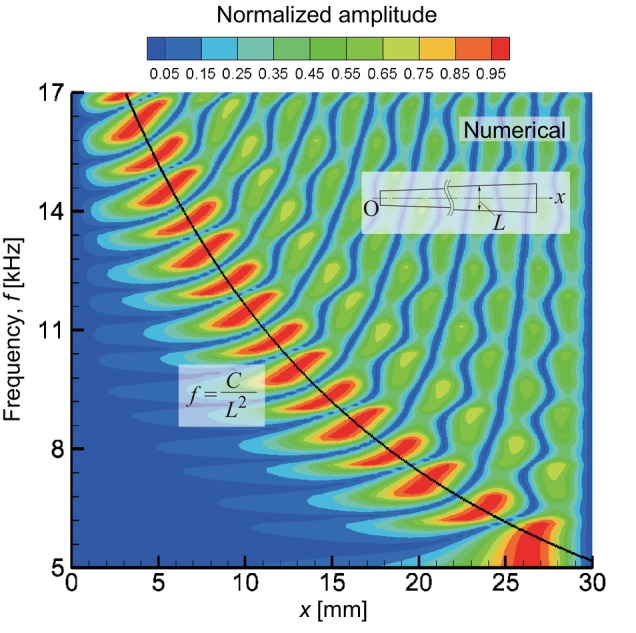
Numerical result of amplitude distribution map as functions of x at y=z=0 mm and applied frequency f, where the amplitude along the *x*‐axis is normalized by the maximum one for each frequency in order to compare the waveforms between various frequencies. The maximum amplitude peaks are fit by a curve of $f=C/L^{2}$ (solid line), where $C=8.28\times 10^{-2}\;m^{2}\;s^{-1}$ and L=x/15+2mm.

First, we computed the eigenfrequency of the fixed membrane for *p*_0_ = 0 Pa. Figure 4 shows the amplitude distributions of some eigenmodes. Figure 4a shows the result from the first eigenfrequency of 5.47 kHz, where the maximum amplitude appeared near the lower base of the trapezoidal membrane. At the second frequency of 6.25 kHz, as shown in Figure 4b, the maximum amplitude point shifted to the upper base side, where a node appeared along the *x*‐axis. Figure 4c shows the third mode, where three peaks appeared along the *x*‐axis and the maximum one was near the upper base. With increasing applied frequency, the maximum amplitude point moved from the lower base to the upper base ends. Figure 4d shows a result from an eigenfrequency of 16.8 kHz, where the maximum peak approached the upper base the most. That is, this shape of the membrane distinguished the eigenfrequency from 5.47 to 16.8 kHz by the maximum amplitude points. The number of peaks that translocate with the eigenfrequency indicates the frequency resolution of the membrane. In addition, the amplitude distributions were extracted along the *x*‐axis at y=z=0mm for applied frequencies from 5 to 17 kHz with an interval of 0.1 kHz. For the forced oscillations, by sweeping the applied frequency with an amplitude of $p_{0}=2.0\times 10^{-4}\;\mathrm{Pa}$ (20 dB SPL), the resonant frequencies were numerically determined at the maximum amplitudes. Figure 5 shows an amplitude map as functions of the x position and applied frequency. Normalizing the amplitude distributions along the *x*‐axis by the maximum values for each frequency, the waveforms could be compared between various frequencies. As a result, it was found that the maximum amplitude peaks were on a curve that was proportional to $1/L^{2}$, which corresponded to the theoretical form expressed by Equation (A5) in the Appendix. Resulting from Equation (A5) and *E* = 12 GPa in accordance with Equation (13), a fixed end beam of length L theoretically shows $f=4.38\times 10^{-2}/L^{2}\;\mathrm{Hz}$. In the numerical result, the maximum amplitude peaks were on a curve of $f=C/L^{2}$ with L=x/15+2mm, where C was a fitting parameter that resulted in $C=8.28\times 10^{-2}\;m^{2}\;s^{-1}$. It is indicated that the eigenfrequency resulting from the trapezoidal‐shape membrane is analogous to the assembly of individually separated single beams of length $L\left(x\right)$. A difference in the numerator between the numerical result and the theoretical model is caused by the different morphologies and disagreement of Young's modulus between the bulk and thin film. Vibrations along the *y*‐axis seem to be sufficiently decoupled from the *x*‐axis, and therefore, the resonant frequencies are successfully analyzed based on separated single beams. As reported in previous studies [11, 34], this is a reason that WKB approximations were successfully applied to the vibration analysis of the basilar membranes.

### 3.2 Experimental Measurement of Resonant Frequencies in Air

**FIGURE 6:**
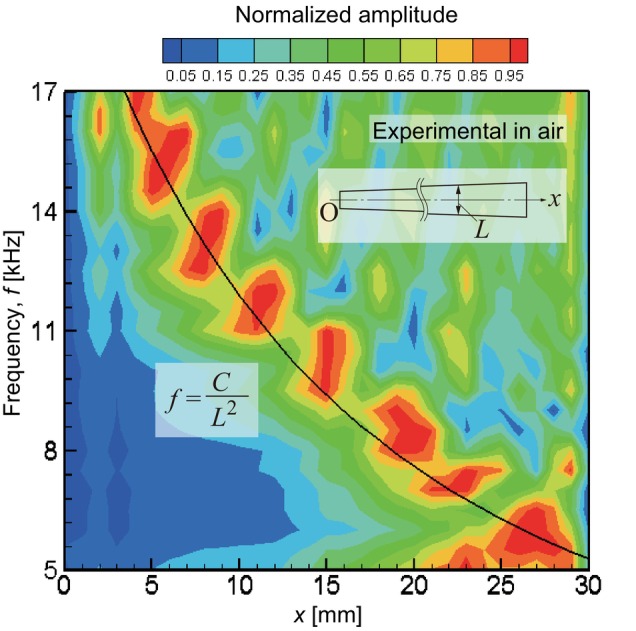
Experimental result of normalized amplitude distribution measured in air along the *x*‐axis at y=z=0mm as functions of x and frequency in the same manner as Figure 5. The maximum amplitude peaks are fit by $f=C/L^{2}\;\mathrm{kHz}$ (solid line), where $C=8.46\times 10^{-2}m^{2}\;s^{-1}$ and L=x/15+2mm.

Using the experimental apparatus shown in Figure 1, vibration characteristics of a trapezoidal membrane were measured in air. Figure 6 shows experimentally observed resonant frequencies presented in the same manner as Figure 5. It was previously found that the amplitudes measured with the LDV system had 5% errors [28] and that the standard deviation of the amplitude peak positions was within 10%. The present experimental result showed a similar trend to the numerical result, in which the maximum amplitude distribution was represented by Equation (A5) in Appendix. The maximum amplitude peaks were on a similar curve shown in Figure 5 with a coefficient of $C=8.46\times 10^{-2}\;m^{2}\;s^{-1}$. It was found that the frequencies from 5 to 17 kHz were identified along the longitudinal direction in the membrane of 30 mm in length. The experimental result showed reasonable agreement with the numerical model shown in Figure 5. On the other hand, the distribution of the peaks along the fitted curve appeared to be coarser in the experimental result than in the numerical result. The number of peaks in the experimental result was about half that compared to the numerical result. It was suggested the numerical model predicted the fine structure in the actual system that might be clarified by higher resolution measurements in the future.

### 3.3 Resonant Frequencies of the Trapezoidal PVDF Membrane in Liquid

**FIGURE 7:**
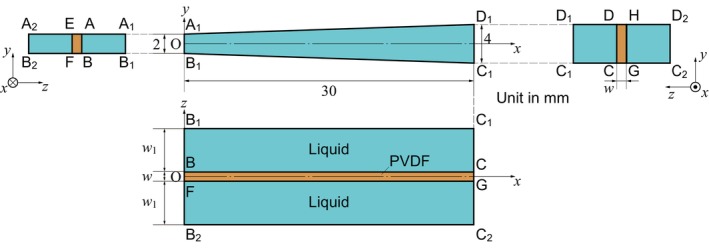
Schematic diagram of a numerical model in which a trapezoidal PVDF membrane immersed in liquid is fixed at the side walls. A uniform stress $p_{0}\sin\left(2\mathrm{\pi ft}\right)$ is applied to surface ABCD. Herein, we set w=52μm and $w_{1}=6\;\mathrm{mm}$, not to scale.

Next, vibrations of the basilar membrane were generated in liquid, and the resonant frequencies were measured using the experimental setup in Figure 1. In the theoretical model, vibrations of the basilar membrane are expressed by Equation (1), which is coupled with sound pressure in liquid, as expressed by Equation (11). Boundary conditions are schematically explained in Figure 7. We set

(18) $$\begin{array}{c} p=0\;\mathrm{on}\;A_{1}{\mathrm{ABB}}_{1},\;B_{1}{\mathrm{BCC}}_{1},\;C_{1}{\mathrm{CDD}}_{1},\;D_{1}{\mathrm{DAA}}_{1},\\ \;{\mathrm{EA}}_{2}B_{2}F,\;{\mathrm{FB}}_{2}C_{2}G,\;{\mathrm{GC}}_{2}D_{2}H,\;\text{and}\;{\mathrm{HD}}_{2}A_{2}E \end{array}$$

(19) $$\frac{\partial p}{\partial z}=0\;\mathrm{on}\;A_{1}B_{1}C_{1}D_{1}\;\text{and}\;A_{2}B_{2}C_{2}D_{2}$$

(20) $$\zeta_{x}=\zeta_{y}=\zeta_{z}=0\;\mathrm{on}\;\text{AEFB},\;\text{BFGC},\;\text{CGHD},\;\text{and}\;\text{DHEA}$$

**FIGURE 8:**
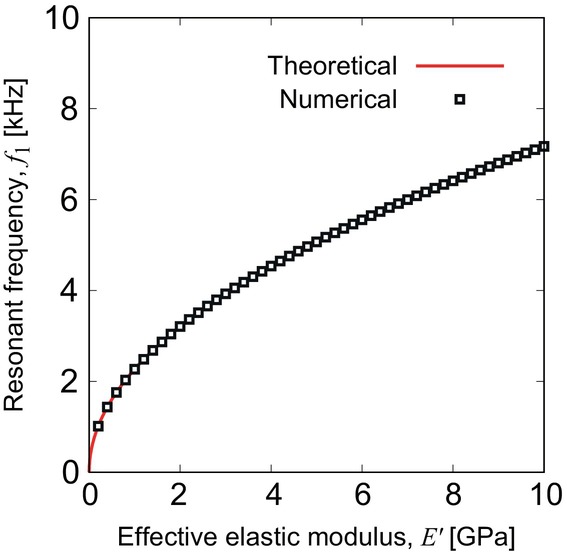
Numerical result of the first‐resonant frequency $f_{1}$ as a function of effective elastic modulus $E^{\prime }$, fitted with the theoretical line that is proportional to $\sqrt{E^{\prime }}$.

**FIGURE 9:**
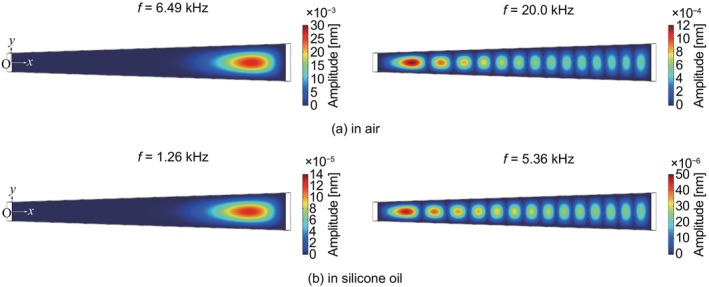
Numerical result of amplitude distributions of (a) the first frequency of $f_{1}=6.49\;\mathrm{kHz}$ and the frequency of 20.0 kHz where the maximum peak was at the nearest to the upper base, computed in air; (b) $f_{1}=1.26\;\mathrm{kHz}$ and 5.36 kHz in the same manner as (a), computed in silicone oil, where $E^{\prime }$ was set to 8.5 GPa.

**FIGURE 10:**
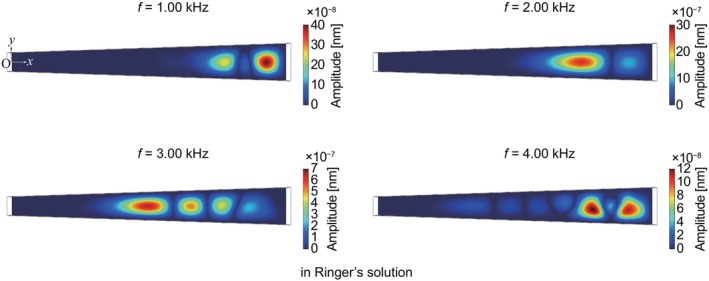
Numerical result of amplitude distributions of 1.00, 2.00, 3.00, and 4.00 kHz, calculated in Ringer's solution for $E^{\prime }=8.5\;\mathrm{GPa}$ by applying a 20 dB pressure difference on the membrane surface.

**FIGURE 11:**
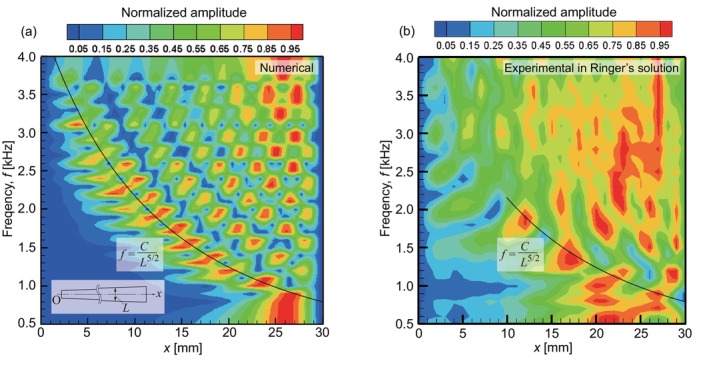
Comparison between (a) numerical and (b) experimental results of normalized amplitude distributions in Ringer's solution, presented in the same manner as Figures 5 and 6, respectively. In both panels, the solid line is expressed as $f=25L^{-\frac{5}{2}}\;\mathrm{kHz}$, where L=x/15+2mm.

Furthermore, the interface between the membrane and the liquid was always contacted during vibrations, satisfying the boundary condition of Equation (12) on ABCD and EFGH. To investigate the resonant frequency in air, physical properties were set to $\rho_{f}=1.20\;\mathrm{kg}\;m^{-3}$, $\mu =1.81\times 10^{-5}\;\mathrm{Pa}\cdot s$, $\mu_{b}=0\;\mathrm{Pa}\cdot s$, and $c_{0}=340\;m\;s^{-1}$. The computational domains were filled with air. For the PVDF membrane, we set $\rho_{m}=1.78\times 10^{3}\;\mathrm{kg}\;m^{-3}$ and E as given by Equation (13). For $p_{0}=2.0\times 10^{-4}\;\mathrm{Pa}$ (20 dB SPL), the resonant frequencies were obtained between 4.14 and 15.0 kHz. On the other hand, previously reported experimental results, measured in air, were between 6.6 and 19.8 kHz [11]. The difference between the present numerical result and the experimental data is caused by the physical properties based on the bulk material. To adjust the numerical data to the experimental data, we propose to modify the elasticity tensor as follows:

(21) $$E=E^{\prime }\left(\begin{array}{cccccc} 1 & 1/2 & 9/38 & 0 & 0 & 0\\ 1/2 & 1 & 9/38 & 0 & 0 & 0\\ 9/38 & 9/38 & 6/19 & 0 & 0 & 0\\ 0 & 0 & 0 & 9/38 & 0 & 0\\ 0 & 0 & 0 & 0 & 7/38 & 0\\ 0 & 0 & 0 & 0 & 0 & 9/38 \end{array}\right)\;\mathrm{GPa}$$

where each component is normalized by the (1,1) element of the elasticity tensor in Equation (13), 3.8 GPa, and parameterized by the multiplier $E^{\prime }$. Varying $E^{\prime }$, the first‐resonant frequency was evaluated and we obtained Figure 8. It was found that the resonant frequency of the first mode, $f_{1}$, was proportional to $\sqrt{E^{\prime }}$, which reflected the theoretical model expressed by Equation (A5) in Appendix, where we defined $E^{\prime }$ as the effective elastic modulus. In this curve, the resonance occurred between 6.49 and 20.0 kHz, when we set $E^{\prime }=8.5\;\mathrm{GPa}$, as shown in Figure 9a. On the other hand, the amplitude tended to be extremely small as a result of fluid–structure interaction in the limitation of a linearized small‐amplitude model. Hereafter, we develop an argument focusing on the frequency selectivity and the amplitude maximum position. For $E^{\prime }=8.5\;\mathrm{GPa}$, this system was simulated in a liquid. Considering a silicone oil, physical properties were set to $\rho_{f}=8.73\times 10^{2}\;\mathrm{kg}\;m^{-3}$, $\mu =1.75\times 10^{-3}\;\mathrm{Pa}\cdot s$, $\mu_{b}=2.50\mu \;\mathrm{Pa}\cdot s$, and $c_{0}=931\;m\;s^{-1}$, where the bulk viscosity coefficient $\mu_{b}$ was treated as an empirical parameter. Although the $\mu_{b}$ of silicone oil has not yet been determined in experiments, we referred to water and other liquids in the literature [42]. Physical properties of some fluids used in this study are summarized in Table 2. Resulting from the computations, we obtained the first‐resonant frequency at $f_{1}=1.26\;\mathrm{kHz}$, which was drastically reduced compared with that in air, as shown in Figure 9(b). This value is in quantitative agreement with the experimental value of 1.4 kHz [11]. The longitudinally highest resonance was found at 5.36 kHz, which was also in reasonable agreement with the experimental value of 4.9 kHz [11]. In the presence of standing waves in the membrane, a difference in the mass density of fluids was more effective on the resonant frequency than the viscosity. It was confirmed that the resonant frequency depended on the density of fluids, but was not seriously changed by the viscosity, although the details of numerical results were omitted here. In such a small‐amplitude vibration, the shear stress caused by the viscosity and the velocity distribution might not work on the membrane surfaces. In experiments, the determination of the highest resonant frequency seemed to be difficult because the amplitude distribution tended to be small with increasing an applied frequency for a constant sound pressure level, and the maximum peaks appeared near the upper base of the trapezoid. This was a possible reason why the experimental value of the highest resonant frequency became lower than the numerical result. As a result, the present method demonstrated that specific materials, having a Young's modulus different from the bulk, were adjusted with experimental data measured in air once, and then the vibration in liquid could be simulated and reproduce the experimental results. Thus, this method is expected to be available to predict experimental results obtained from various liquids. Herein, we measured the resonant frequency of the trapezoidal membrane immersed in Ringer's solution that was an electrolyte solution, the pH and osmotic pressure of which were prepared to be the same as the lymphatic liquid in the human body. We also performed a numerical analysis in which the physical properties of Ringer's solution were set to $\rho_{f}=1.05\times 10^{3}\;\mathrm{kg}\;m^{-3}$, $\mu =1.02\times 10^{-3}\;\mathrm{Pa}\cdot s$, $\mu_{b}=2.79\mu \;\mathrm{Pa}\cdot s$, and $c_{0}=1480\;m\;s^{-1}$, where $\mu_{b}$ was reasonably determined from the value of water [42]. The trapezoidal membrane was fixed between the liquid layers of $w_{1}=6\;\mathrm{mm}$ in thickness, which was determined from the actual thickness used in the experimental system. As mentioned above, the thickness of the membrane was w=52μm, which was equivalent to that in the present experiment. Figure 10 shows the numerically evaluated amplitude distributions to the applied frequency of f=1.00, 2.00, 3.00, and 4.00 kHz. This result suggested that the amplitude distribution might not show the frequency selectivity to the off‐resonance frequencies, because the amplitude maximum position did not necessarily appear at the leading position of the peak series. In this case, resonant frequencies appeared near 2.00 and 3.00 kHz as 2.05 and 3.09 kHz, respectively, and then the maximum peaks were at the leading position. On the other hand, the frequencies of f=1.00 and 4.00 kHz were far from the resonance, and therefore, peaks appeared near the lower base end. The amplitude map resulting from the experiment and the numerical analysis is compared in Figure 11. In both cases, it was found that the resonant frequencies were distinguished by the membrane in the range from 0.5 to 4 kHz. As shown in Figure 11a, the maximum amplitude peaks tended to appear along a curve proportional to $L^{-5/2}$, which was different from the case in the air. In a previous study, Lim et al. [33] suggested that the resonant frequency of a boundary‐fixed beam in liquids could be expressed as $\omega^{2}=\frac{D_{22}}{\rho_{f}}{\left(\frac{\pi }{L}\right)}^{5}$, where ω was the angular frequency and $D_{22}$ was the corresponding membrane stiffness. In the theoretical framework, the L dependency on the resonant frequency might become stronger than that in air due to the inertia of liquids as mentioned above. On the other hand, the trapezoidal shape caused to induce some amplitude peaks in addition to the maximum one. In the experimental result, as shown in Figure 11b, this trend was also observed, especially in the range from x=10 to 30 mm. The amplitude tended to broadly distribute along the *x*‐axis and also appeared near the lower base of the trapezoid due to off‐resonance, as indicated in Figure 10. It was clarified that the amplitude at small L tended to be suppressed in liquid with increasing the resonant frequency. That is, the frequency selectivity of the trapezoidal membrane was degraded in Ringer's solution. This trend was similar in both experimental and numerical results. It is suggested that the frequency selectivity of the membrane is constrained in liquid compared with in air. A difference in the resonant frequency between the air and liquids depends on their physical properties and eigenfrequencies. According to Equation (A12) in Appendix, the eigenfrequencies of silicone oil and Ringer's solution are 2.7 and 4.4 times larger than that of the air, respectively, where the eigenfrequency of the air is 28.3 kHz for L=6mm (or 5.67 kHz for L=30mm). In the fluid–structure interaction, the trapezoidal membrane tends to be the most resonant with the air, among the three type fluids. On the other hand, Ringer's solution, the eigenfrequencies of which are far from those of the membrane, rather works to suppress the vibration as a damper. For practical applications, the artificial cochlea will be used in lymphatic liquids like a Ringer's solution, and the present device need to be improved to realize the wide‐range frequency selectivity. Frequency matching between the membrane and liquid in the fluid–structure interaction is important to extract preferable functions for the artificial cochlea. In a recent study [28], a control system to amplify the most prominent peaks, suppressing others, has been tested. It is expected that the present system will be improved by applying the amplification of the amplitude of leading positions corresponding to the resonant frequencies.

**TABLE 2:** Physical properties used in the FEM analysis for liquid–structure interactions.

	Air	Silicone oil	Ringer's solution
$\rho_{f}\;\left(\mathrm{kg}\;m^{-3}\right)$	1.20	$8.73\times 10^{2}$	$1.05\times 10^{3}$
$c_{0}\left(m\;s^{-1}\right)$	340	931	1480
$\mu \;\left(\mathrm{Pa}\cdot s\right)$	$1.81\times 10^{-5}$	$1.75\times 10^{-3}$	$1.02\times 10^{-3}$
$\mu_{b}/\mu$	0	2.50	2.79

### 3.4 Resonant Frequency Analysis of Artificial Basilar Membrane of Guinea Pig

**FIGURE 12:**
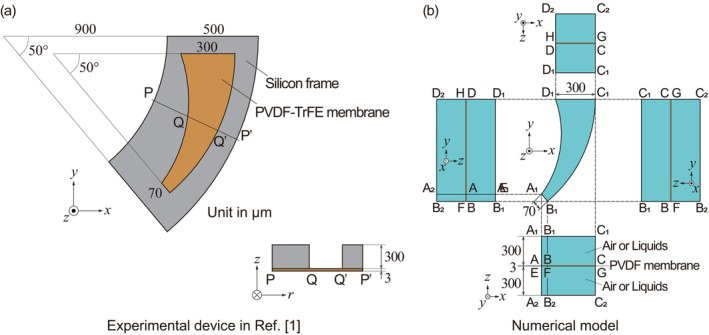
(a) Illustration of the experimental device of the artificial basilar membrane for guinea pig cochlea, which was made of a PVDF‐TrFE membrane fixed on the silicon frame, as described in the literature [1]. (b) Numerical model of the present FEM analysis for the resonant frequency analysis of PVDF membrane that has the same dimension as the experimental device shown in (a).

**FIGURE 13:**
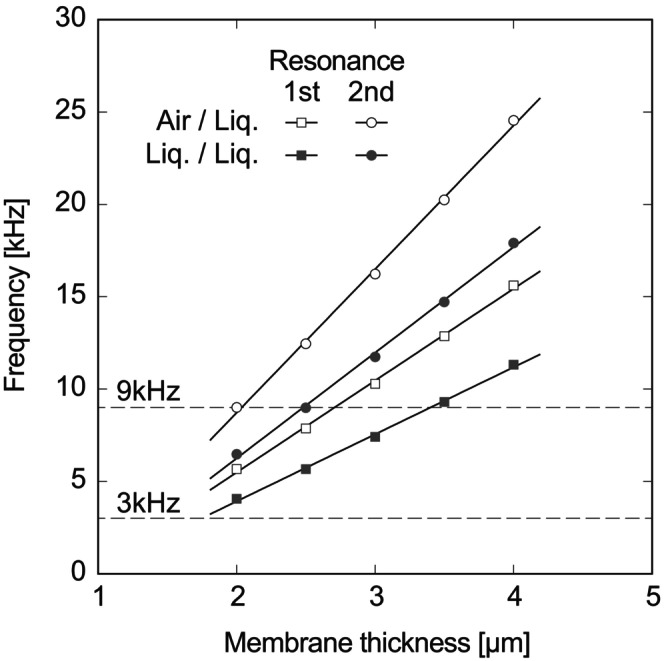
Numerical results of the first‐ and second‐resonant frequencies as a function of the PVDF membrane thickness, resulting from the fluid–structure interaction, where “Air/Liq.” and “Liq./Liq.” in the legend mean that the membrane was placed between the air and Ringer's solution (open square and open circle) and between Ringer's solutions (closed square and closed circle), respectively. The first‐ and second‐resonant frequencies of 3 and 9 kHz experimentally measured [1] are also shown by dashed lines, respectively.

**FIGURE 14:**
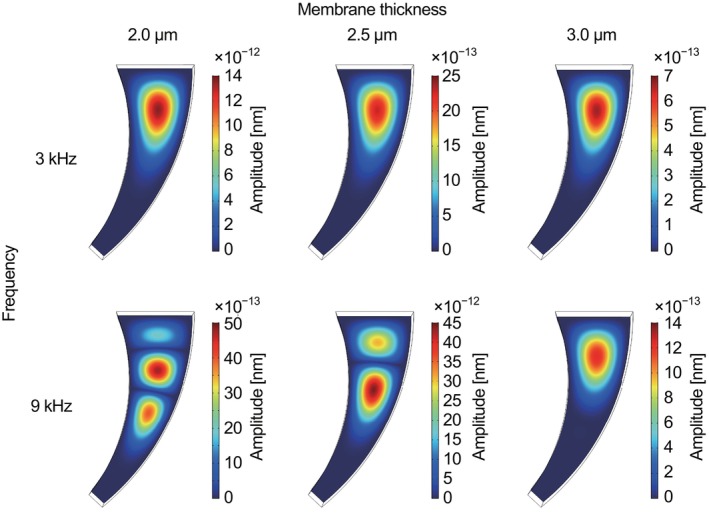
Numerical results of amplitude distributions in the PVDF membrane of 2.0, 2.5, and 3.0 μm thick fully immersed in Ringer's solution, responding to an applied pressure difference of 20 dB SPL with the frequency of 3 and 9 kHz [1]. Each membrane showed the aspect of the first mode oscillation to an applied frequency of 3 kHz, and on the other hand, the result was varied for 9 kHz application.

In a previous study [1], a mimic of the basilar membrane of a guinea pig was fabricated using photolithography and reactive ion etching. An artificial membrane made of PVDF‐TrFE, which was designed for the basilar membrane of the guinea pig, as shown in Figure 12a, was measured. In this study, the resonant characteristics of the artificial membrane were investigated considering the fluid–structure interaction in the FEM analysis. Figure 12b shows an illustration of the numerical model, whose dimension reproduces the artificial basilar membrane of the guinea pig in Figure 12a. The thickness of the PVDF membrane was set to 3 μm, which was estimated in the experiment [1]. In the previous result, the resonant frequency was determined at 3 and 9 kHz for the first‐ and second‐resonance, respectively, using the artificial membrane implanted in a guinea pig cochlea. Although these frequencies coincided with the resonance of the basilar membrane of the guinea pig, the mechanism has not yet been clarified. Here, it was assumed that the elasticity of PVDF‐TrFE could be linearly scaled by that of PVDF. The curved shape of the PVDF membrane that mimics the basilar membrane of the guinea pig was much smaller than the former trapezoidal membrane. In the numerical model, the resonant frequency of this shape was evaluated in air and Ringer's solution, considering the fluid–structure interaction with similar boundary conditions for the case of the trapezoidal membrane (Equations 12 and (18), (19), (20)). An oscillating pressure difference of 20 dB SPL was applied in the normal direction on the top surface of the membrane. Based on the elasticity tensor of bulk PVDF represented by Equation (13), the resonant frequency in the air was determined as 85.1 kHz that was much larger than the corresponding experimental data of 37 kHz previously measured (unpublished data). The elasticity tensor was scaled by a coefficient of $E^{\prime }=0.72\;\mathrm{GPa}$ in Equation (21) that reproduced the experimental result of 37 kHz in air. Numerical results of the first‐ and second‐resonant frequencies of the PVDF membrane for guinea pig cochlea are shown in Figure 13. For the computational conditions, two situations were examined, in which the PVDF membrane was partly or fully immersed in Ringer's solution. It was clear that the resonant frequencies were reduced in viscous liquids. The dependency of the membrane thickness on the resonant frequency was also investigated. Both the first‐ and second‐resonant frequencies showed linearity for the membrane thickness between 2 and 4 μm. The resonant frequencies were proportional to the membrane thickness. This result was reasonable compared with the theoretical model of an elastic beam whose ends were fixed, as described in Appendix A, where the fraction of the second momentum of the area to the cross‐section was proportional to the square of the thickness, and the frequency was proportional to the square root of it. In Figure 13, typical values of 3 and 9 kHz for the first‐ and second‐resonant frequencies [1] were also indicated by dashed lines, respectively. Some of the computational results appeared near the experimental data, although they did not fully agree with the frequency of 3 and 9 kHz. Especially for a membrane of 2.5 μm thick fully immersed in Ringer's solution, the first‐resonant frequency was at 5.66 kHz and the second mode appeared at 8.99 kHz which was the closest to the experimental value. Figure 14 shows amplitude distributions of the membranes of 2.0, 2.5, and 3.0 μm thick fully immersed in Ringer's solution, resulting from the computations for the forced oscillations by applying an acoustic pressure difference of 20 dB SPL with the frequencies of 3 and 9 kHz. It was found that each membrane showed the aspect of the first mode to the frequency of 3 kHz. For the applied frequency of 9 kHz, amplitude distributions varied with the membrane thickness. The membrane of 2.0 μm thick showed three peaks in which the amplitude was the largest at the central peak. The aspect of the third‐mode oscillation seemed to appear because the second‐resonant frequency of the 2.0 μm thick membrane was 6.47 kHz that was lower than 9 kHz. The membrane of 2.5 μm thick whose second‐resonant frequency was 8.99 kHz prominently responded to the applied frequency. There were two peaks and the largest one shifted to the narrower end of the curved shape compared with the first mode. On the other hand, the second‐mode vibration was not excited in the membrane of 3.0 μm thick, which was evaluated as 11.75 kHz. In a previous experiment [1], it was suggested that the resonant frequencies of 3 and 9 kHz were induced by the resonance of the basilar membrane of a guinea pig cochlea and that the artificial piezoelectric membrane also well responded to the vibration of the basilar membrane. The present result from the 2.5 μm thick membrane was in reasonable agreement with the previous experiment [1], although the membrane thickness might be a little bit underestimated compared with the experimental evaluation of 3 μm.

In addition to the resonant characteristics of the trapezoidal PVDF membrane, the artificial basilar membrane for guinea pigs could reproduce experimental results based on the FEM analysis considering the fluid–structure interaction. Consequently, quantitative evaluations remaining in previous studies were proceeded in this study.

On the other hand, the present model assumed a small limit of amplitude, and therefore, the quantitative evaluations were limited to the resonant frequencies. The amplitude was possibly discussed with respect to qualitative evaluations, such as vibration modes and displacement distributions. For the quantitative discussion about the amplitude, nonlinear models of self‐excited vibration in continuum media are required and may enable us to represent the nonlinear functions of cochlear implants [27, 29, 43].

## 4 Conclusions

In the present study, focusing on the resonant characteristics of the basilar membrane for the artificial cochlea, the frequency selectivity of a trapezoidal piezoelectric membrane that mimicked guinea pig cochlea was numerically investigated compared to the experimental results [1, 11]. Owing to the tapered shapes, oscillating pressure differences externally applied were separately sensed at different positions along the membrane. Considering the fluid–structure interaction, details in the frequency selectivity of the PVDF membranes in liquid were clarified as follows:

- The resonant frequency range sensed by the trapezoidal PVDF membrane in the air was clearly decreased in liquid, which quantitatively reproduced the experimental results.
- In the numerical analysis considering the fluid–structure interaction, scaled Young's modulus adjusted to experimentally measured resonant frequencies in the air could well reproduce the resonant characteristics of the membranes in liquid. It was suggested that frequency matching between the membrane and liquids in the fluid–structure interaction systems had a possibility to extend the frequency selectivity available for the development of the artificial cochlea.
- According to the proposed method, resonant frequencies of the artificial piezoelectric membrane for guinea pig cochlea, observed at 3 and 9 kHz in the animal test, were also reproduced.

Consequently, the present numerical procedure will effectively contribute to optimize the resonant characteristics of the artificial basilar membrane in various liquids. This will be an important technique for the development of artificial micro‐electromechanical devices in which the physical properties of materials are different from the bulk and unknown in specific situations.

## Ethics Statement

The authors have nothing to report.

## Consent

The authors have nothing to report.

## Conflicts of Interest

The authors declare no conflicts of interest.

## Figures and Tables

**FIGURE A1:**
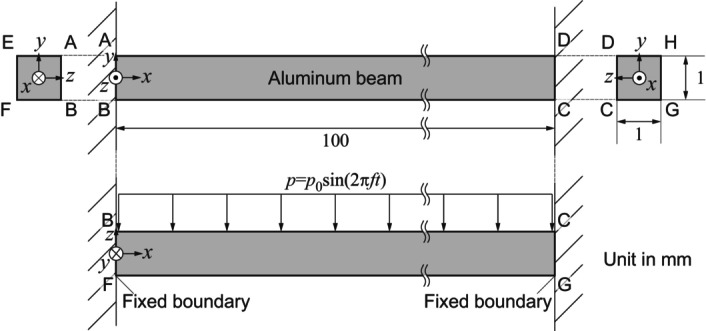
Schematic illustration of an aluminum beam, which has a square cross‐section of $1\times 1\;{\mathrm{mm}}^{2}$ and a length of 100 mm, fixed at both ends of AEFB and CGHD. A pressure difference of $p_{0}\sin\left(2\mathrm{\pi ft}\right)$ is applied in the z direction.

**FIGURE A2:**
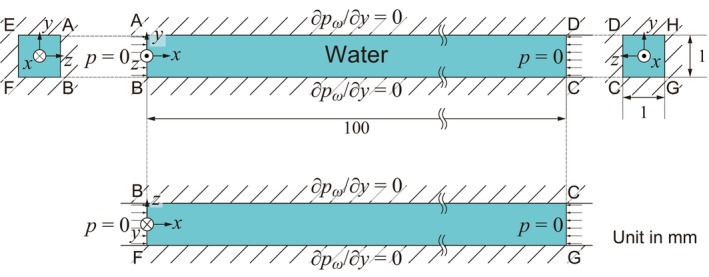
Schematic illustration of water in a square duct, which has a $1\times 1\;{\mathrm{mm}}^{2}$ and a length of 100 mm, fixed at both ends of AEFB and CGHD.

## Data Availability

The data that support the findings of this study are available from the corresponding author upon reasonable request.

## Appendix A

### A.1 Validation of the Numerical Analysis

Before carrying out vibration mode analysis of a trapezoidal PVDF membrane in liquid, the numerical accuracy is verified for the elastic body motion. As shown in Figure A1, an aluminum beam that has a square cross‐section of $1\times 1\;{\mathrm{mm}}^{2}$ and a length of 100 mm is fixed at both ends. A uniform oscillating pressure difference of $p_{0}\sin\left(2\mathrm{\pi ft}\right)$ was applied on a plane of ABCD in the z direction, where $p_{0}$ was a constant that proportionally affected the amplitude, and f was the externally applied frequency. Considering the physical properties of aluminum, we set $\rho_{m}=2.70\times 10^{3}\;\mathrm{kg}\;m^{-3}$, E=70.0GPa, and η=0.33. The boundary conditions were set to

(A1)
$$\sigma_{z}=p_{0}\sin\left(2\mathrm{\pi ft}\right)\;\mathrm{on}\;\text{ABCD}$$

(A2)
$$\frac{\partial \zeta_{z}}{\partial z}=0\;\mathrm{on}\;\text{GHEF}$$

(A3)
$$\frac{\partial \zeta_{y}}{\partial y}=0\;\mathrm{on}\;\text{BFGC}\;\text{and}\;\text{DHEA}$$

(A4)
$$\zeta_{x}=\zeta_{y}=\zeta_{z}=0\;\mathrm{on}\;\text{AEFB}\;\text{and}\;\text{CGHD}$$

where the resonant frequency was evaluated by solving Equation (1). As a result, the first‐resonant frequency was $f_{1}=523.8\;\mathrm{Hz}$. On the other hand, in the theoretical model, the first‐resonant frequency of the both‐end fixed beam is given as follows:

(A5)
$$f_{1t}=\frac{22.37}{2\pi L^{2}}\sqrt{\frac{\mathrm{EJ}}{\rho_{m}A}}$$

where L is the beam length, A is the cross‐section area, and J is the second momentum of area. Referring to the physical properties of aluminum, the theoretical resonant frequency results in $f_{1t}=523.3\;\mathrm{Hz}$. The difference between the numerical and theoretical results is 0.13%, which is quite small, and both of the results are in good agreement.

### A.2 Sound Pressure Propagation in Liquid

Next, the computational accuracy of sound pressure propagation in a water tube is evaluated. As shown in Figure A2, water is in a square cross‐section duct. The physical properties of water were set to $\rho_{f}=1.00\times 10^{3}\;\mathrm{kg}\;m^{-3}$, $c_{0}=1.48\times 10^{3}\;m\;s^{-1}$, and $\mu =1.0\times 10^{-3}\;\mathrm{Pa}\cdot s$. Here, we adjusted the value of $\mu_{b}=2.79\mu$ that was required to reproduce the eigen frequency of the water tube and was reasonably determined compared with previously reported data [42]. The importance of liquid bulk viscosity for vibration problems was reported in the literature [32, 37]. Considering a vibration state, $\mathrm{\delta p}\left(x,y,z,t\right)=\delta p_{\omega }\left(x,y,z\right)\exp\left(\mathrm{i\omega t}\right)$ is substituted in Equation (11), where ω and $\delta p_{\omega }$ are the angular frequency and its amplitude, respectively. Then, Equation (11) is transformed as follows: [42]

(A6)
$$c_{c}^{2}\Delta^{2}\delta p_{\omega }-\omega^{2}\delta p_{\omega }=0$$

where

(A7)
$$c_{c}^{2}=c_{0}^{2}\left(1+\mathrm{i\omega}\frac{\nu \tilde{V}}{c_{0}^{2}}\right)$$

Boundary conditions are set to

(A8)
$$\delta p_{\omega }=0\;\mathrm{on}\;\text{AEFB}\;\text{and}\;\text{CGHD}$$

(A9)
$$\frac{\partial \delta p_{\omega }}{\partial y}=0\;\mathrm{on}\;\text{BFGC}\;\text{and}\;\text{DHEA}$$

(A10)
$$\frac{\partial \delta p_{\omega }}{\partial z}=0\;\mathrm{on}\;\text{ABCD}\;\text{and}\;\text{GFEH}$$

where Equation (A6) is numerically solved, and the first‐resonant frequency is obtained as $f_{1}=7.40\times 10^{3}\;\mathrm{Hz}$. To verify this numerical result, we theoretically analyze Equation (A6) for the one‐dimensional problem in the x‐axis. The boundary conditions of the duct of length L are set to

(A11)
$$\mathrm{\delta p}\left(0,t\right)=\mathrm{\delta p}\left(L,t\right)=0$$

where the analytical solution is obtained, and the first‐resonant frequency results in

(A12)
$$f_{1t}=\frac{1}{2L}\sqrt{c_{0}^{2}-\frac{1}{4}{\tilde{V}}^{2}\nu^{2}{\left(\frac{\pi }{L}\right)}^{2}}$$

Substituting the physical properties of water and the length of L=100mm in Equation (A12), we obtain $f_{1t}=7.4\times 10^{3}\;\mathrm{Hz}$. This value is in good agreement with the aforementioned numerical result.

As mentioned above, vibrations of elastic bodies and sound pressures in water were numerically analyzed with high accuracy. Based on these results, the resonant frequency of PVDF basilar membranes is evaluated in the main text.
